# Investigation of erosion-corrosion failure of API X52 carbon steel pipeline

**DOI:** 10.1038/s41598-023-42556-6

**Published:** 2023-11-22

**Authors:** Mahmoud T. Abdu, Waleed Khalifa, Maiada S. Abdelrahman

**Affiliations:** Department of Metallurgical Engineering, Faculty of Engineering, Cairo University, Giza, 12613 Egypt

**Keywords:** Engineering, Materials science

## Abstract

A failure analysis of API X52 steel pipeline was conducted. The investigation included complete material characterizations using tensile and hardness testing, optical microscope, SEM, and EDS. The main failure occurred in the downstream pipe located near the welded joint at the elbow outlet instead of elbow which was interesting. The main mechanism of failure was found to be erosion-corrosion mechanism that caused breakdown of the protective FeCO_3_ film, thinning of the downstream pipe, and finally failure. It is believed that the erosion-corrosion was induced by sand impingement due to turbulent flow that was promoted by sudden change in the flow cross section between the elbow inlet and upstream pipe and poor welding quality of joint at the elbow outlet.

## Introduction

Oil and gas pipelines are long distance means of transportation that are used throughout the world for transporting liquid (mainly oil) or gas from their sources to refineries, distribution sites and purchasers or consumers^1^. The material selection for the pipeline is critical, and it is required to carefully investigate the operating environment for proper choice^2^. Other factors besides environment can affect the material selections such as water content, gases present, pressure and temperature, flow rate, pH, and design life. However, most oil and gas pipelines all over the world are fabricated from carbon steel. This is related to the fact that carbon steel is available, relatively cheap, and has good mechanical properties and excellent weldability. Furthermore, the corrosion resistance of the carbon steel can be modified by coating, lining, cladding and chemical inhabitation. Many carbon steel grads were used for oil and gas pipelines such as grades A, B, or C according to ASTM specifications A-53 and A106 and API 5L standard^3^. Unfortunately, the high strength low alloy API 5L grades (X42-X100) pipelines are reported as the most grades susceptible to failure^4^.

Oil and natural gas pipelines offer a safe and cost-effective method of transportation, but any pipeline leakage or damage can lead to catastrophic results^5^. Failure of pipelines can be due to different metallurgical mechanisms such as manufacturing defects, third party damage, and corrosion^6,7^. However, it has been reported that corrosion is the predominant root cause for failure of pipelines in many countries^8^. Corrosion-induced damage causes gradual decrease in pipe integrity that promotes failure. Consequently, this failure results in additional cost and unfavorable accidents such as explosions and fires which lead to severe environmental issues^9^. Corrosion failure of the pipeline is mainly due to the presence of CO_2_, H_2_S, H_2_O, bacteria, sand, and organic acids forced into the fluid inside the pipelines. Also, the gas obtained from the crude oil production mainly consists of methane, ethane, and hydrocarbons^10^. This gas becomes a wet gas due to insufficient dehydration which leads to contents of liquid hydrocarbons and water, acid gases (ex. CO_2_ and H_2_S), and dissolved ions (ex. Cl^−^ and Ca^2+^)^11,12^. Under certain conditions of temperature and pressure these contents can severely attack the inner wall of piping equipment^13^.

Several corrosion forms were reported to produce failure in the oil and gas pipelines. Erosion-corrosion is considered one of the most common corrosion forms that is found in oil and gas production plants and pipelines. It mainly depends on the interaction between solid particles, corrosive media, and material surface^14^. Erosion-corrosion is a form of tribo-corrosion material loss mechanism that damages both the passive film and the base metal beneath^15^. Erosion-corrosion involved a combined effect of mechanical and electrochemical processes that act and affect each other^16^. Thus, the material loss is caused by chemical dissolution at the surface that removes the protective film and mechanical erosion due to fluid flow and/or impingement of the particles on the pipe wall. In some cases, mechanical erosion is enhanced by electrochemical corrosion. Unfortunately, the risk of erosion in gas pipelines is high because the conveying velocities in gas pipelines are higher compared to other fluids^17,18^. Also, the erosion of the C-Mn steel is enhanced by 2–4 times by the corrosion in aqueous CO_2_ environment^19^. Further, the material loss due to erosion-corrosion is much higher than that caused by pure mechanical erosion or pure electrochemical corrosion^20^.

Under certain operating conditions including CO_2_ environment, a protective film of iron carbonate (FeCO_3_) is formed on the surface of carbon steel pipeline. However, the formation of such protective film depends on several parameters such as temperature, CO_2_ partial pressure, pH value, flow velocity, and supersaturation^21^. Under temperature greater than 60 °C, pH higher than 5, supersaturation greater than zero, and higher CO_2_ partial pressure, the protective film is formed^22^. However, high flow velocity reduces the rate of FeCO_3_ precipitation by reducing the supersaturation level at the metal surface^21^. Moreover, the protective film is deposited on the steel surface at different rates according to temperature and solubility product^23^. Unfortunately, sand particles in the flow can impact the pipeline surface causing erosion of the protective film. Consequently, corrosion of the parent metal is permitted through uncovering the active metal surface. Thus, this type of erosion-corrosion is considered a threatening form of metal loss due to the synergistic effect of erosion and corrosion^21^. Therefore, prediction and control of the erosion-corrosion attack become vital for facilities’ safety and cost considerations^24^.

However, until now the interaction between erosion and CO_2_ corrosion in some of the engineering applications such as elbows installed in the pipelines is poorly understood^25^. Also, the related research work on CO_2_ erosion-corrosion of carbon steel due to gas–solid flow in aqueous environment are currently still less investigated. Thus, it becomes necessary for safe production to carefully study the mechanism of failures and do countermeasures based on the operating conditions^26^.

In this article we are going to present a detailed failure analysis of API X52 high strength low alloy steel natural gas production pipeline. The pipeline includes the upstream pipe, elbow, and the downstream pipe. The failure analysis will focus on comprehensive study for the pipeline system history service, the possible root causes for failure, the probable failure mechanisms, and the future corrective actions to avoid such events. The investigation will include complete material characterizations using destructive mechanical testing (tensile and hardness testing), optical microscope (OM), scanning electron microscope (SEM), and energy dispersive X-ray spectroscopy (EDS).

## Experimental methods

### Material

A failed high strength low alloy carbon steel natural gas pipeline with an outer diameter of 8 inches was supplied. The supplied materials of the gas pipeline were upstream pipe, downstream pipe, and elbow (smooth and round 90-degree type). The pipeline was supplied as grade API X52 high strength low alloy carbon steel. The pipeline was installed for the transportation of natural gas that consisted of approximately 96% methane and 2.3% ethane (see Table 1 for gas analysis). The flow rate was 1.8 MMSCFD (Million Standard Cubic Feet per Day). The flow velocity was approximately 22 m/s. The working pressure in the downstream portion was 95 bar and the operating temperature was 61.5 °C. The carbon steel pipeline was subjected to failure twice since the startup of the line in 2015. Unfortunately, the installation documents which include installation and welding procedures were not available for the authors.

**Table 1:** The gas analysis for the pipeline.

Comp., wt%	N_2_	C1	CO_2_	C2	C3	i-C4	n-C4	i-C5	n-C5	C6	C7	C8	C9 +	H_2_O, ppmv
Samp.1	0.037	90.4	1.97	4.05	1.58	0.52	0.45	0.22	0.16	0.25	0.19	0.12	0.05	75
Samp.2	0.038	90.3	1.91	4.03	1.58	0.52	0.46	0.23	0.17	0.28	0.23	0.16	0.07	160

The chemical analysis of the failed pipeline was identified by optical emission analysis as shown in Table 2. Chemical analyses were performed for the upstream pipe, elbow, and downstream pipe. The resultant chemical composition for all the components conforms to grade X52 according to the standard API 5L^27^.

**Table 2:** The chemical composition of the failed pipeline.

Elements	C	Mn	Si	P	S	Ni	Cr	Cu	Mo	V	Nb	Ti
Downstream pipe	0.104	0.87	0.26	0.008	0.003	0.12	0.15	0.18	0.02	0.06	0.00	0.00
Elbow	0.21	0.89	0.30	0.019	0.004	0.13	0.16	0.16	0.06	0.00	0.00	0.00
Upstream pipe	0.14	1.03	0.34	0.012	0.004	0.03	0.05	0.08	0.02	0.01	0.00	0.00
API 5L-X52^27^	0.28 max	1.4 max	–	0.03 max	0.03 max	–	–			Nb + V + Ti ≤ 0.15%		

### Visual examination

Visual inspection was performed using naked eye and macroscopic examination to evaluate the fractured surface of the failed pipeline. The evaluation was performed for the downstream pipe, upstream pipe, and elbow. Also, the welded joints between the elbow outlet and downstream pipe and between the elbow inlet and upstream pipe were examined to investigate the soundness of the welded joints.

### Microstructure characterization

The microstructure characterization of the failed pipeline involves qualitative and quantitative analysis of fracture surface topography and possible manufacturing defects. The microstructure characterization of the fractured surface was performed using optical microscope, scanning electron microscope (SEM), and semi-quantitative energy dispersive X-ray spectroscopy (EDS). The surface was prepared for microstructural examination using griding, polishing, and etching. Nital etchant solution was prepared by mixing 100 ml ethanol and 2 ml nitric acid^28^. The sample was immersed in the solution for 30 s to reveal grain boundaries.

Both of the macro and microstructures are basic steps of the conventional failure analysis procedure. The macrostructure investigation demonstrated both the location of failure and the main features of failure. The macrostructure examination is one of the most important steps that are used to determine the damage mechanism. As for the microstructure, it is used for more investigation of the pipeline system (upstream pipe, elbow, and downstream pipe) by comparing the microstructure at its different parts and introducing closer look for the failed portion. The microstructure controls the metal properties and performance. Thus, both are essential to reach the root cause of failure.

### Mechanical tests

Two mechanical tests were performed. The tensile test was conducted to investigate the tensile properties of the downstream pipe, elbow, and upstream pipe. The tensile test was conducted according to the standard ASTM E8/E8M-22 and the tensile properties were examined to be confirmed with that of carbon steel pipeline of grade X52 according to API 5L^27,29^. However, the minimum specified elongation (A_f_) expressed in percentage and accepted as per by API 5L standard is calculated according to the following equation^27^:

$${A}_{f}=C\frac{{A}_{xc}^{0.2}}{{U}^{0.9}}$$

where C is a constant that equals to 1940 for using SI units in the calculations, A_xc_ is the applicable tensile test piece cross-sectional area in mm^2^, and U is the specified minimum tensile strength in MPa. The minimum specified elongation is calculated based on both the measured cross-sectional area of the tensile test sample and specified minimum tensile strength and then compared to the measured elongation.

The Vickers hardness test is used to measure the hardness of the downstream pipe, elbow, and upstream pipe and it was conducted according to standard ASTM E92-17^30^.

## Results and discussion

High strength low alloy carbon steel API X52 pipeline was erected for the transportation of natural gas. A failure analysis was acquired to identify the root cause of the failure, its mechanism, and the corrective actions to avoid such an accident. The failure analysis investigation included visual inspection, mechanical testing and complete microstructural characterization.

### Visual inspection

**Figure 1:**
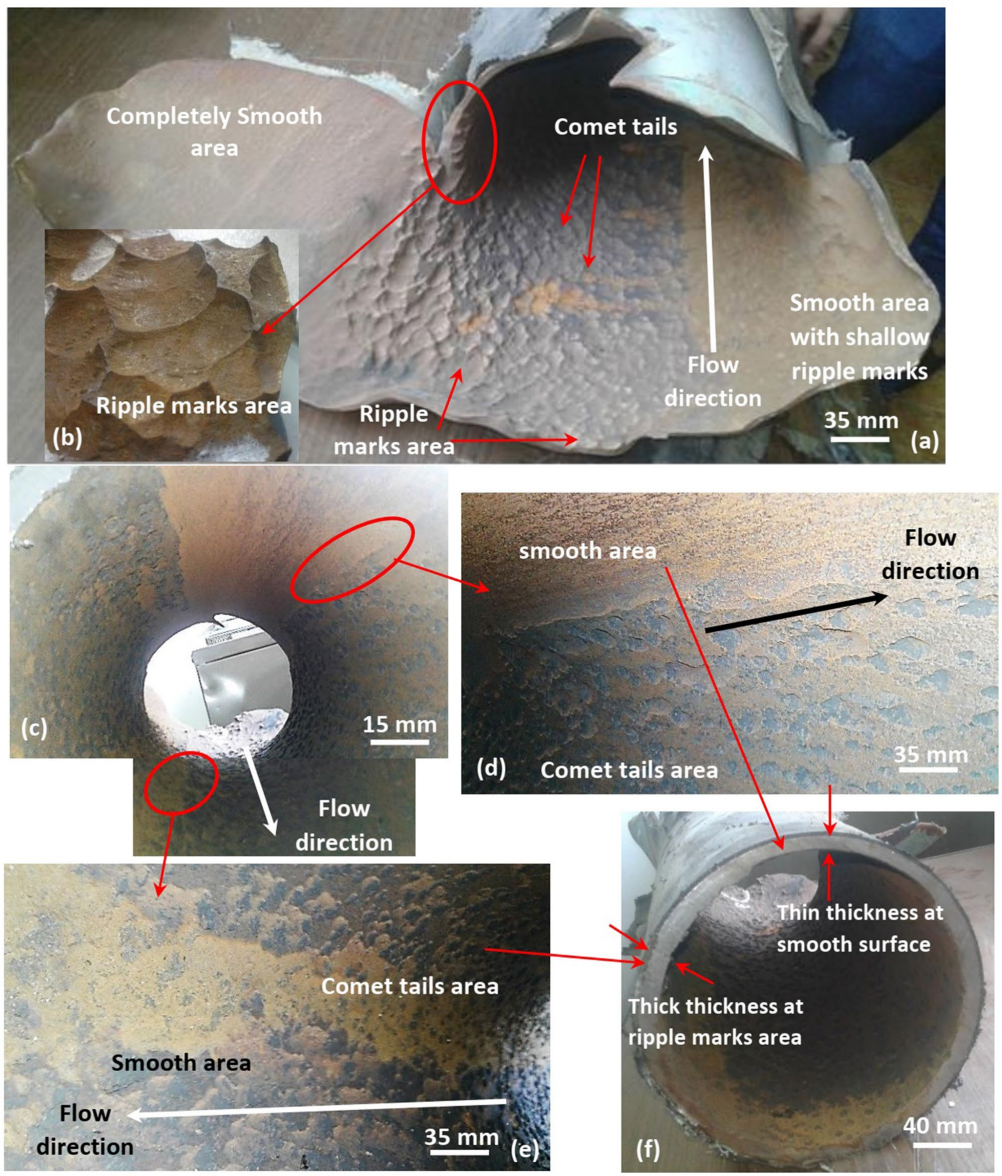
Fractography of the failed downstream pipe (**a**) internal surface shows smooth area and ripple marks area, (**b**) closer look at the ripple marks area, (**c**) internal surface at 0.6 m distance from the failed side, (**d**) and (**e**) smooth and comet tails areas appear at different positions on the circumference, and (**f**) thickness variation corresponding to the comet tails area and smooth area.

**Figure 2:**
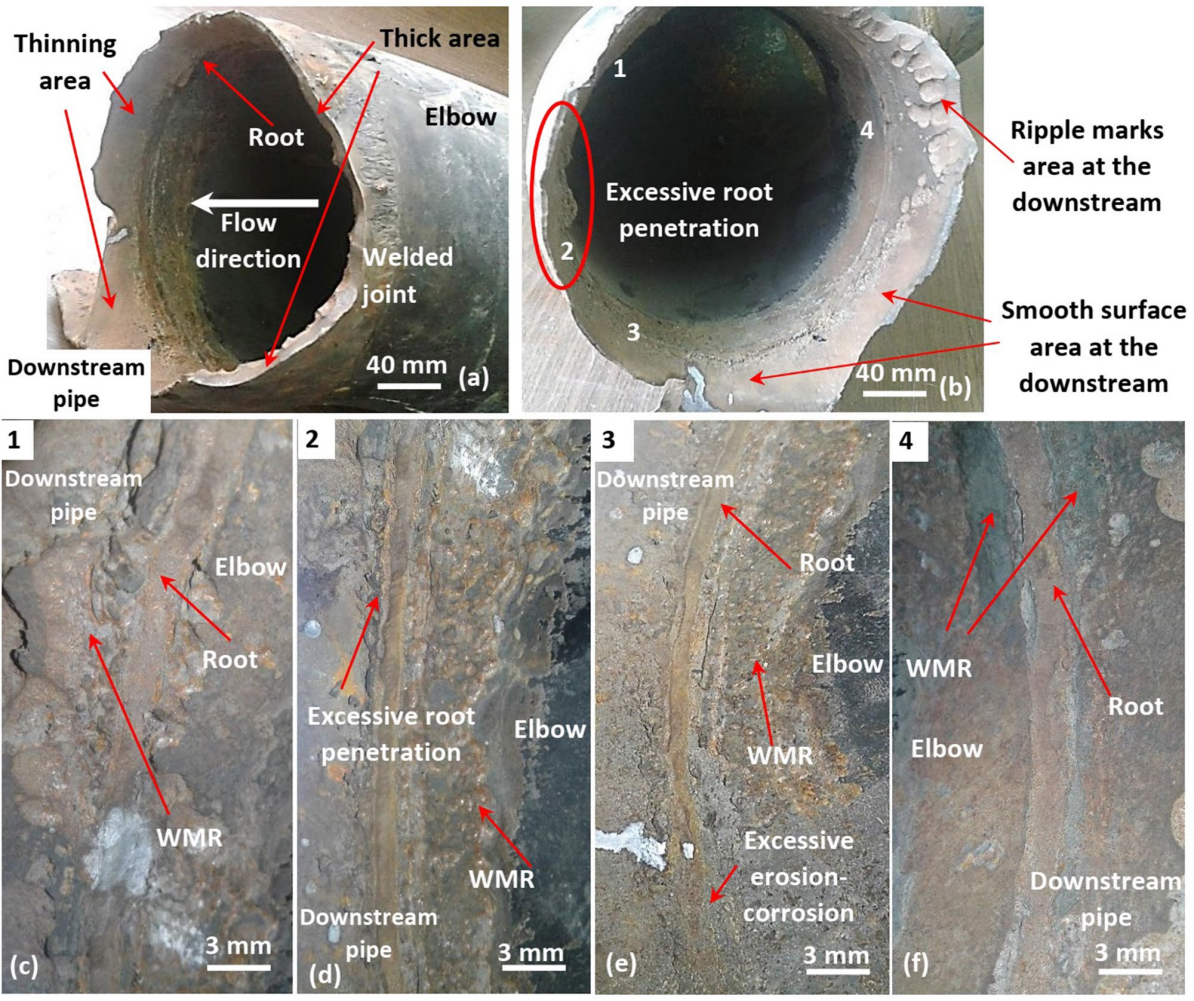
Fractography of the welded joint between the elbow and downstream pipe. (**a**) The failure location at the downstream pipe just near the welded joint, (**b**) front view for the welded joint demonstrates the excessive root penetration, and (**c**–**f**) closer look for the root at the points 1 to 4 located on (**b**).

**Figure 3:**
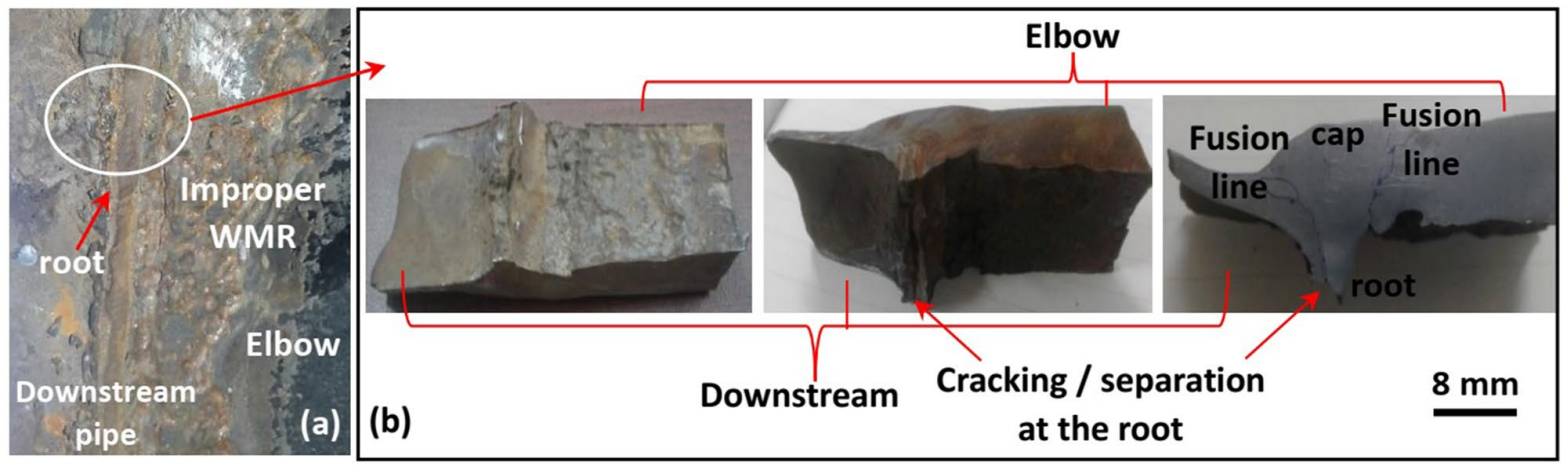
Macrosample for excessive root penetration. (**a**) location of the macrosample and (**b**) root with cracking or separation along its line at the severely damaged downstream pipe side.

**Figure 4:**
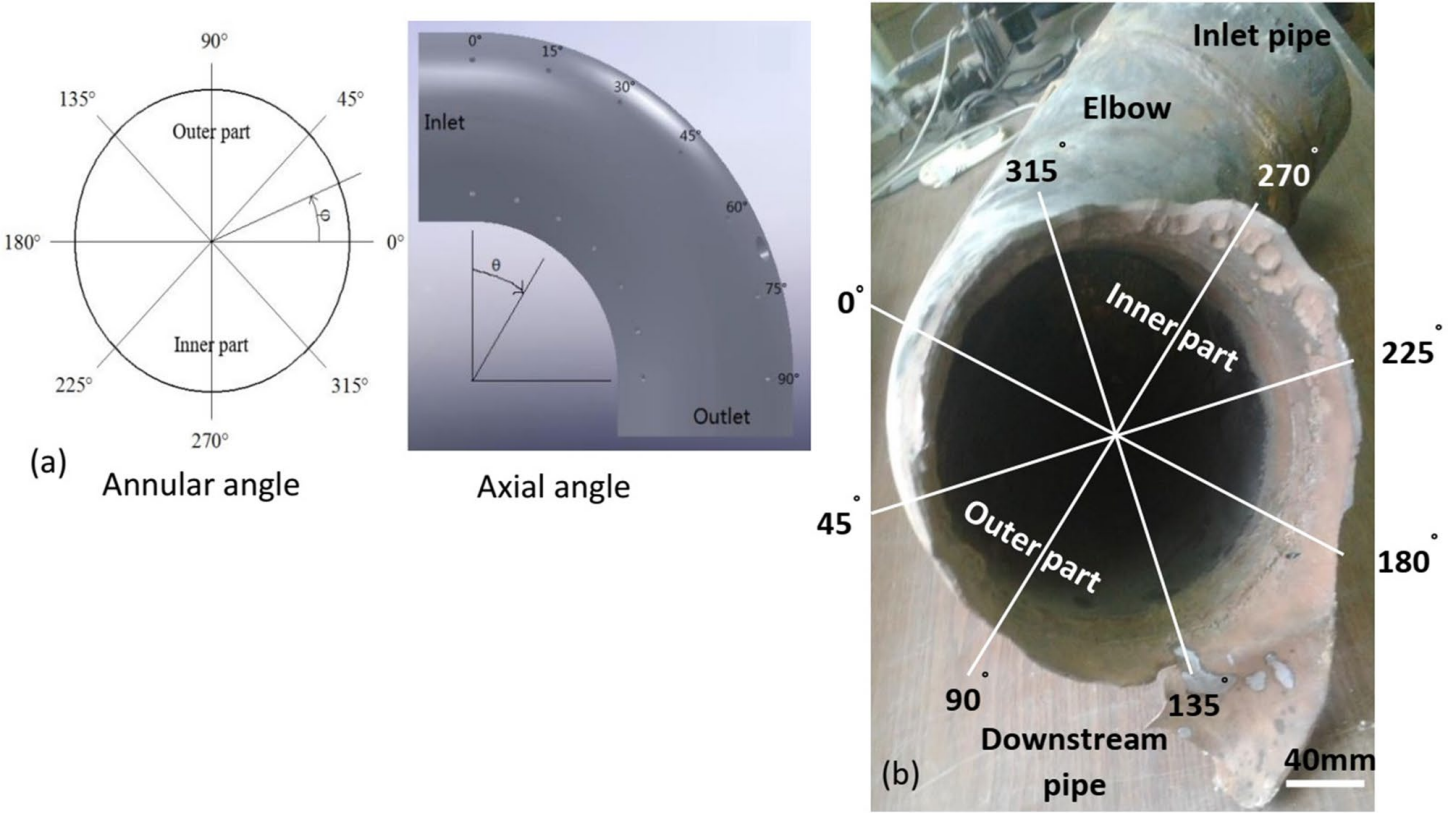
Failure position according to annular angle and axial angle. (**a**) Schematic diagram showing annular and axial angles^49^ and (**b**) annular angles distribution on the elbow.

**Figure 5:**
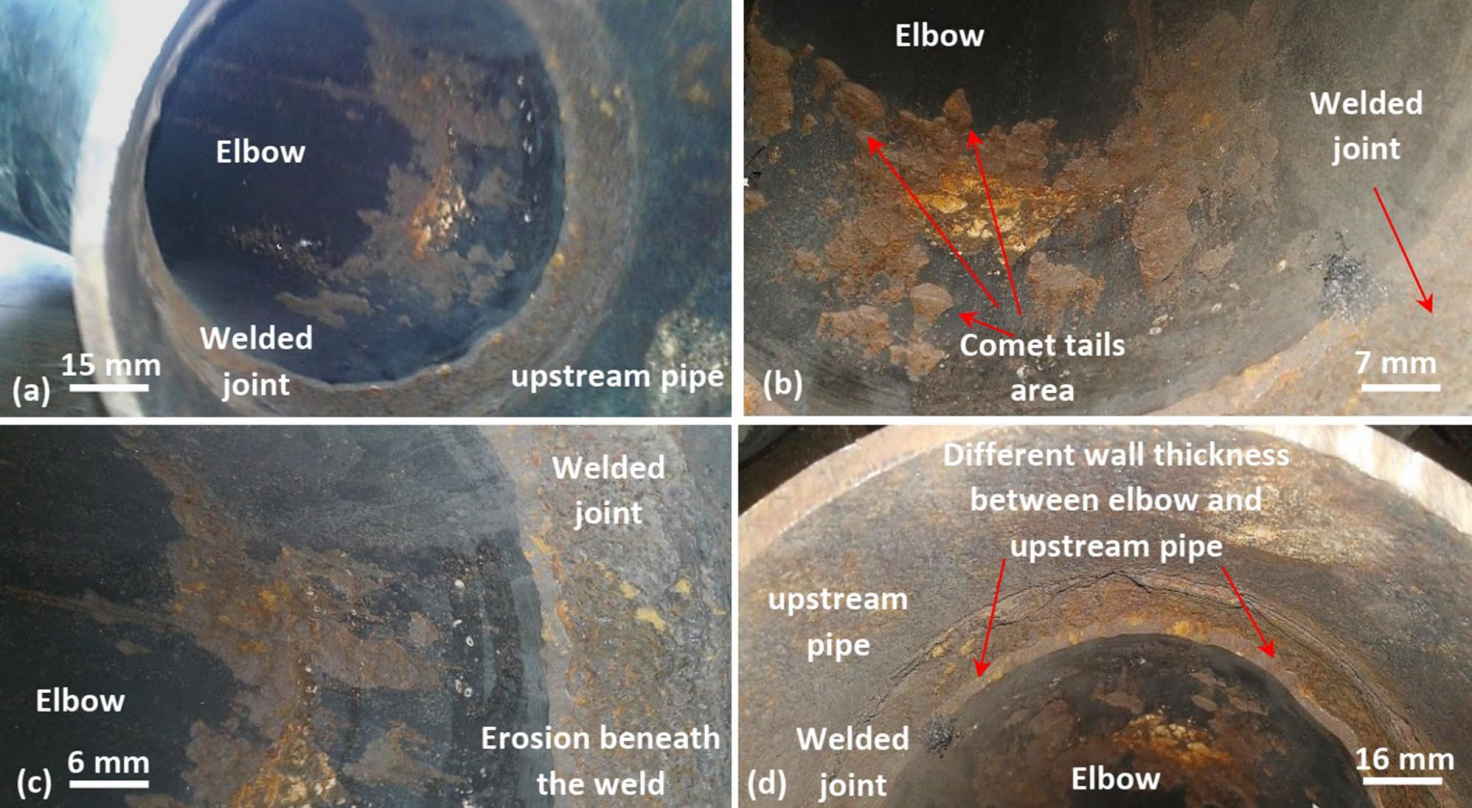
Fractography of the welded joint between the elbow and upstream pipe. (**a**) general view for the welded joint, (**b**) comet tails area in the elbow adjacent to the welded joint, (**c**) erosion beneath the weld, and (**d**) different wall thickness between the elbow and upstream pipe.

**Figure 6:**
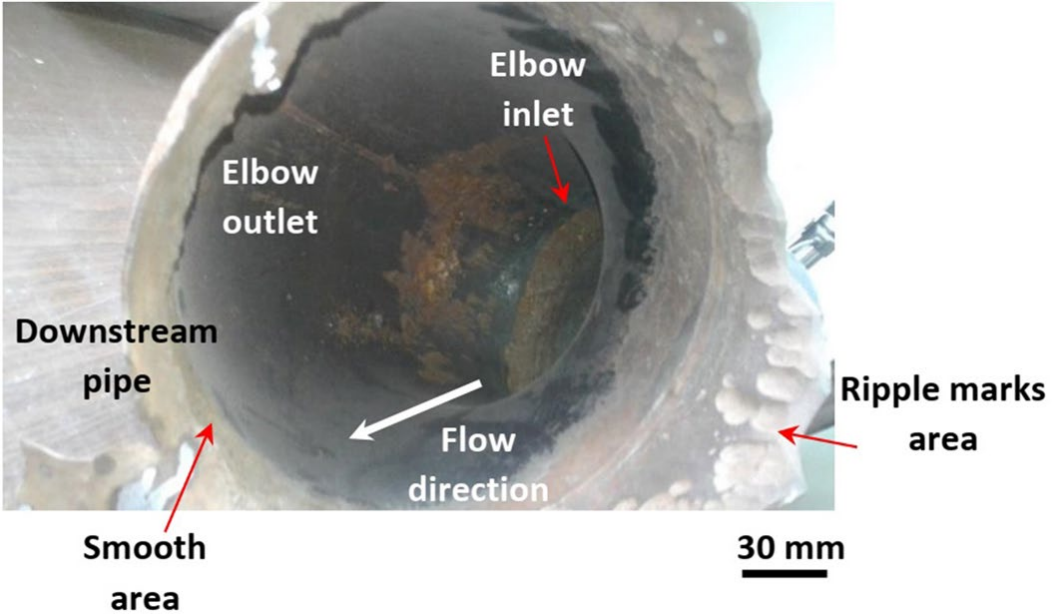
The severe erosion-corrosion damage at the elbow inlet in line with the most-thinned area (smooth area).

A visual inspection of the failed API X52 carbon steel gas pipeline was performed. Figures 1, 2, 3, 4, 5 and 6 demonstrate the fractography of the failed pipeline at five zones: downstream pipe, welded joint between downstream pipe and elbow outlet, elbow, welded joint between the elbow inlet and upstream pipe, and upstream pipe. Obviously, the main failure occurred at the downstream pipe in the area adjacent to its welded joint with the elbow outlet as shown in Figs. 1 and 2. Figure 1a–e shows that the inner surface of the downstream pipe consists of two distinguished areas along the length of the pipe, smooth (abraded) surface area and deeply grooved surface area. The fractured surface (see Fig. 1a–e) illustrates two types of transition in the fractography features, namely, longitudinal and transverse. The longitudinal transition occurred at the deeply grooved area along the length of the pipe. The deeply grooved area demonstrates transition from ripple marks area at the fractured side (see Fig. 1a and b) to comet tails area along pipe length (see Fig. 1c–e). As for the transverse transition, the fracture morphology varies from deeply grooved area to smooth area with shallow ripple marks and then completely smooth area (see Fig. 1a). The smooth area with shallow ripple marks is present at the right side of the deeply grooved area while the completely smooth area is at the left side. The transition appears when the two sides (i.e., the right and left) are folded and reconnected together again. All the illustrated features are indications for the CO_2_ erosion-corrosion mechanism, which is reported to appear in the form of abrasion, ripple marks, horseshoes, comet tails and dinosaur footprint^31,32^.

As mentioned previously erosion-corrosion is caused by a combined effect of the mechanical erosion of metal due to particle impingement or fluid flow and electrochemical process that removes the surface passive layer^17^. Sand is considered the most common and destructive erodent for the pipelines^21^. Silty sand of size smaller than 50 µm can easily enter the pipelines through sand screens and affect the CO_2_ corrosion^33,34^. Thus, the probability for sand erosion due to the presence of sand particles in the flow gas is high.

Figure 1f shows variation in the wall thickness along downstream pipe cross section. The wall thickness dropped over only two years from 8.8 mm to less than 1 mm for the smooth surface area at the failed side (i.e., the corrosion rate is roughly 4 mm/year). The protective layer of iron carbonate (FeCO_3_) preferentially forms on the metal surface in the CO_2_ environment under the conditions of pH > 5 and temperature of 60 °C^22^ which are typical to that of the present case. However, sand impingement could partially or completely remove the protective layer leading to interfacial plastic deformation on the pipe surface, which facilitates crack propagation^35^. Rincon et al.^36^ reported that the corrosion rate is promoted by 2–4 times in CO_2_ environment containing sand with size of 150 µm compared to that with no sand. Hence, the sand impingement imposes erosion and accelerates the CO_2_ corrosion rate and thinning of the pipeline, especially near the failure location. Furthermore, CO_2_ corrosion enhances the erosion rate too by dissolving the work-hardened layer, speeding up the removal of the embedded sand particles and decreasing the surface hardness at the anodic sites which in turn promotes the erosion process^37^. Once the thinning of the wall thickness takes place as the erosion starts, the material bearing capacity sequentially drops leading to shortening of the service life and finally failure^38^. Therefore, CO_2_ erosion-corrosion is considered the main root cause and mechanism for failure in the present failure case.

However, the wall thinning through the circumference of the downstream pipe was inhomogeneous, which is revealed by the difference in the wall thickness at the smooth and deeply grooved areas. The smooth area is thinner than the deep grooved area. This can be related to the turbulent flow that led to instability in the flow velocity, sand impingement, and erosion-corrosion rate through the same cross section.

Turbulent flow was also found to assist both the transverse and longitudinal transitions in the fracture morphology. The turbulent flow promotes the transverse transition through enhancing the variation of the erosion-corrosion rate. Consequently, the ripple marks along the circumference are gradually eroded from the right side of the deeply grooved area to completely disappear at left side.

The longitudinal transition also occurs due to the turbulent flow or varied flow velocity. Xu et al.^39^ reported that comet tails are formed due to erosion-corrosion on carbon steel at the low flow velocity (1 to 3 m/s) of seawater while pits are found at higher flow velocities (5 to 8 m/s). Hence, the comet tails at distance from the failed side in our failure case are formed at the relatively lower flow velocity compared to flow velocity at the fractured side (see Fig. 1a-e). Comet tails are produced by anolyte transportation initiated at tip pits to downstream^39,40^. The comet tails were found to become shorter with the increase of the flow velocity (3 m/s) and longer at lower flow velocity (1.5 m/s)^39,41^. Further, the density of the pits increased with the increase in the flow velocity^39^. Thus, the comet tails in the present case become shorter and change into pits with the increase in the flow velocity. Moreover, the increase in the pit density merges the pits into larger pits that are thought to form ripple marks at higher flow velocity at the fractured side.

Figure 2 shows the welded joint between the elbow outlet and the downstream pipe. Obviously, the fracture occurs in the downstream pipe near the welded joint, while the internal surface of the elbow near the downstream side seems unaffected as shown in Fig. 2a and b. This was unpredictable because the effect of erosion particles on elbow is 50 times or more serious than in the straight pipe^42^. Further, Fig. 2a illustrates the wall thinning along circumference of the downstream pipe especially near the fracture. The completely smooth area underwent the most thinning while the ripple marks area maintained some of the wall thickness. This local thinning can be related to the severe erosion induced by the turbulent flow in such areas compared to other at the ripple marks area.

Further investigation for the welded joint was performed to uncover the cause of the turbulent flow. Figure 2b–f shows clear evidence for improper welding procedure that appears in the excessive root penetration, sharp root profile, and improper weld metal reinforcement (WMR) next to the root. Obviously, the welded joint surface is rough which significantly affects the flow stability and correspondingly the erosion rate. The degree of roughness was found to increase the flow instability and turbulence rate, especially close to the surface^43,44^. Also, the erosion wear was reported to increase with the increase in the flow velocity^45^. Thus, surface irregularity along the welded joint promoted both the turbulent flow and erosion wear.

Moreover, the root surface demonstrates a narrow and sharp profile, which can be preferential site for stress concentrations and cracks (see Figs. 2d and 3). Figure 3b shows cracking or separation starting from the sharp edge of the root, and continuing along fusion line between the weld metal and downstream pipe. Stress concentration caused the breakdown of the passive film in the area and induced localized dissolution^46^. Increasing stress concentration accelerated the local corrosion rate which, in turn increased the degree of surface roughness and sharpness and transferred the corrosion morphology from a flatter to a pit^47^. Hence, the sharp profile of the root can be a preferential site for cracking and starting the corrosion damage that is assisted by the turbulent flow induced erosion. Furthermore, this corrosion damage can even promote the formation of ripple marks near the welded joint as they are thought to be formed by merging of the pits.

Weld metal reinforcement next to the root seemed to be introduced to reduce the influence of root erosion induced by the flow (see Fig. 2c–f). Unfortunately, the application of weld metal reinforcement was improper as it had uneven width along circumferential root. Also, it disappeared in some locations, especially next to the root near the most wall thinned area in the downstream pipe (see point 3 in Fig. 2b and e). Further, local circumferential erosion-corrosion groove was observed along circumferential weld near that area. Concludingly, the width of the weld zone along the welded joint between the elbow outlet and downstream pipe was irregular, leading to surface roughness and flow disturbance.

Qiao et al.^48^, reported that the elbow causes a change in the flow pattern which significantly affects the downstream weld. Also, Liu et al.^49^ investigated the effect of flow velocity (from 3.5 to 4m/s) on the erosion-corrosion of 90° horizontal elbow. They found that the maximum erosion-corrosion rate occurred at the elbow outlet with axial angle between 75° to 90° at two positions. The first one was in the outer part at annular angles of 45°, 90°, and 135° and the second was in the inner part at annular angles of 225°, 270°, and 315°. Figure 3 compares the position of failure according to axial and annular angles with Liu et al. findings (see Fig. 4a). Figure 4b demonstrates the annular angles distribution on the elbow at the side of the downstream pipe. Clearly, the most wall thinning in the downstream pipe next to the elbow outlet with axial angle 90° occurred in the outer part at the annular angles between 45° to 180° and at the inner part at angles between 180° to 225°. This shift in the annular angles can be related to the poor quality of the weld joint.

Thus, turbulent flow induced by the elbow and poor welding quality joint led to severe damage at the downstream weld instead of the elbow outlet, which in turn causes the wall thinning and failure at downstream pipe.

Figure 5 shows the welded joint between the elbow inlet and the upstream pipe. Obviously, the elbow inlet underwent severe erosion-corrosion attack in the outer part next to the welded joint as revealed in Fig. 5a–d. Figure 5b demonstrates several comet tails as evidence for the erosion-corrosion attack in the elbow inlet. The comet tails seem to accumulate in the outer part only of the elbow inlet (i.e., no sign for comet tails in the inner part). Further, the girth weld is subjected to a circumferential corrosion groove as shown in Fig. 5a, c, and d. This can be an indication for turbulent flow that led to sand impingement with higher velocity and severe erosion.

The cause of turbulent flow in the elbow could be deduced from the examination of Fig. 5d. Figure 5d demonstrates difference in the wall thickness between the elbow inlet and upstream pipe, which are equal in the outer diameters. The elbow shows wall thickness of 11 mm, while that of upstream pipe is 8.8 mm. Thus, there is sudden change in the flow cross section at the contact area between the elbow inlet and upstream pipe. Hence, the turbulent flow can be easily promoted by such conditions leading to erosion-corrosion damage in the elbow.

Furthermore, Liu et al.^49^ reported that increasing the particle velocity increased their mechanical effect and induced secondary flow. Consequently, different velocity contours in the different cross sections of the elbow were formed leading to different erosion-corrosion rates. Thus, the sudden change in the flow cross section at the elbow inlet caused variation in particles velocity and their impact angle. As a result, different erosion-corrosion effects occurred at the downstream pipe. Those effects were represented in the distinguished areas found in the downstream pipe that were formed due to the turbulent flow and the variation in erosion-corrosion rate. Also, the poor welding quality of the joint between the elbow and downstream pipe could take part in the various effects of the erosion-corrosion in the downstream pipe. Figure 6 emphasizes that conclusion as the severe erosion-corrosion damage induced by higher flow velocity and occurs at the elbow inlet is in line with the most-thinned area (smooth area), where the failure took place at the downstream pipe.

### Microstructure characterization

Complete microstructural characterization was performed on the elbow, downstream pipe, upstream pipe, and the welded joint at the outlet of the elbow to detect microstructural defects, and further investigate root cause of the failure. However, the failure occurred next to the welded joint between the elbow outlet and downstream pipe and so the microstructure investigation was focused only on that weld joint. The microstructure characterization included microstructure examination by optical microscope, SEM examination, and EDX examination.

**Figure 7:**
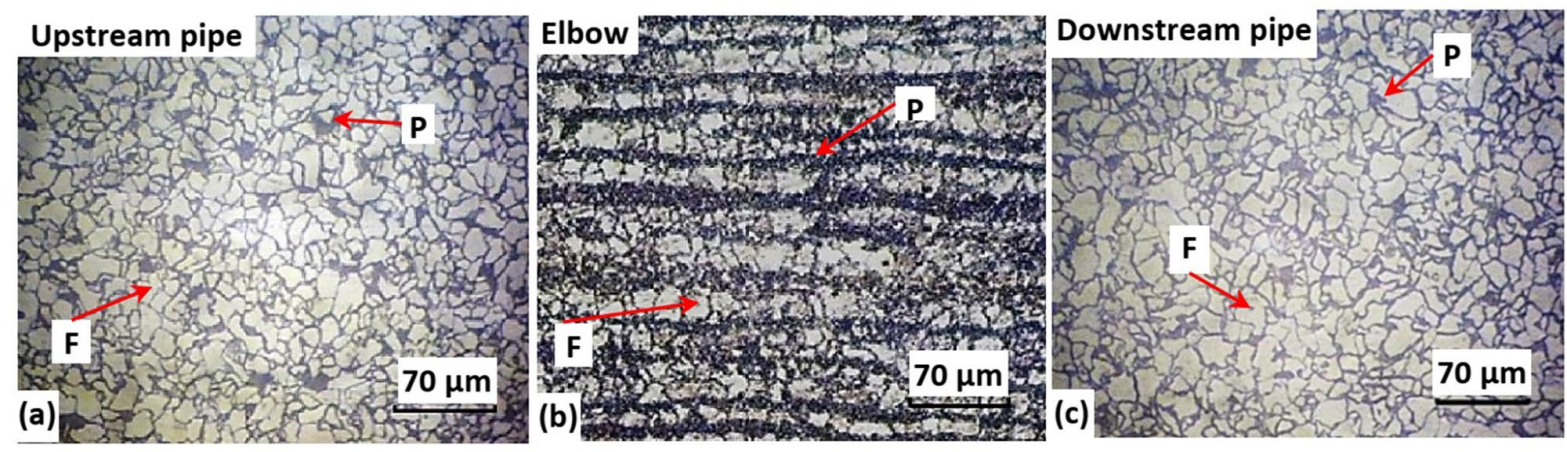
Microstructure of (**a**) upstream pipe, (**b**) elbow, and (**c**) downstream pipe. The bright phase is ferrite (F), and dark phase is pearlite (P). (200×).

**Figure 8:**
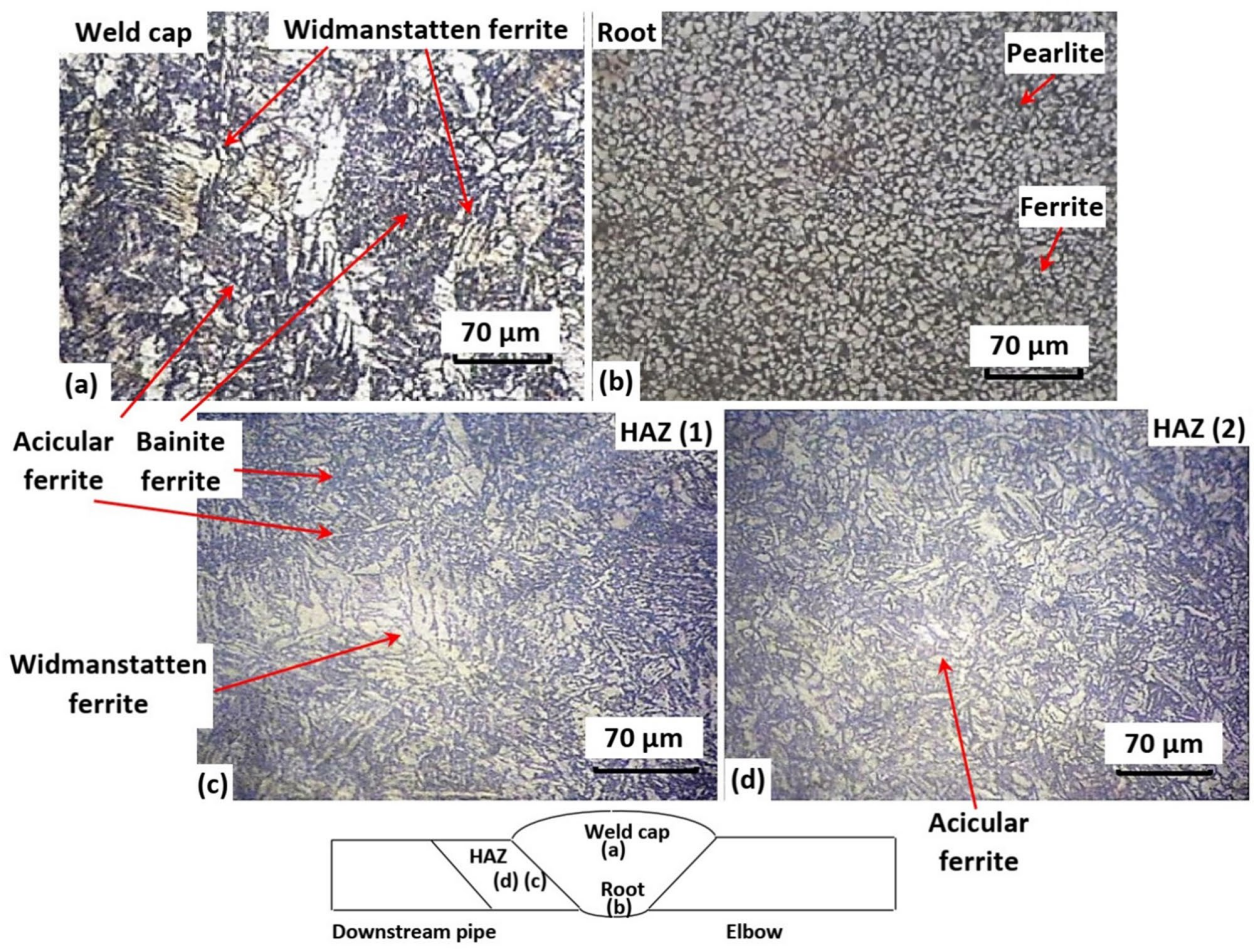
Microstructure of welded joint at different zones: (**a**) weld cap, (**b**) root, (**c**) HAZ of the downstream pipe adjacent to fusion line and (**d**) HAZ of the downstream pipe at fine-grained zone. (200×).

The microstructure was examined using the optical microscope as shown in Figs. 7 and 8. Figure 7 demonstrates the base metal microstructure of upstream pipe, elbow, and downstream pipe. Both upstream and downstream pipes show microstructure of typical normalized API 5L X52 steel, which consists of pearlite colonies distributed over equiaxed ferrite matrix (see Fig. 7a and c)^50^.

It is well known that the pearlite is dark on the bright field optical micrographs, and the ferrite is bright on the same. Why this occurred is explained here. The pearlite phase always appears as dark phase in bright field microscopes due to its laminar structure that reflected less light, while the ferrite appears as bright phase because of its equiaxed form that reflects more light. Similar microstructure of API 5L X52 steel was reported by other authors as referred to reference^50^.

Thus, the bright grains belong to the ferrite phase, while the dark ones are for pearlite structure. The microstructure was normal with no sign of any discontinuities and was accepted as a delivery condition for X52 steel according to the API 5L standard^27^. Figure 7b reveals the microstructure of the elbow, which was a typical ferrite/pearlite banded structure of X52 steel in as-rolled condition. The bright bands are equiaxed ferrite phase, and the dark band are pearlite structure. Although the banded structure causes segregation of the substitutional alloying elements and anisotropy in some of the mechanical properties, but the microstructure is still accepted for the delivery condition according to API 5L standard ^27,51^.

Figure 8 demonstrates the microstructure of different welding zones of the welded joint between the elbow outlet and downstream pipe. The investigated welding zones are the weld metal cap, weld metal root, and heat affected zone (HAZ) of the downstream pipe. The microstructure of the weld metal cap shows normal microstructure of Widmanstatten ferrite at the grain boundaries and acicular ferrite and bainite ferrite at the interior of the grain (see Fig. 8a)^52,53^. On the other hand, the microstructure of weld-metal root reveals a typical equiaxed ferrite matrix with fine pearlite on grain boundaries (dark constituents) (see Fig. 8b). Such microstructure is considered as normalized weld structure that is obtained due to the reheating effect induced by the multi-passe procedure^54^. Figure 8c and d illustrates the microstructure of downstream HAZ adjacent to the fusion line (at coarse-grained HAZ) and at distant (i.e., inside the fine-grained HAZ zone), respectively. The microstructure of HAZ adjacent to the fusion line shows coarse Widmanstatten ferrite at the grain boundaries and fine acicular ferrite and bainite ferrite inside the grain^53,55^ (see Fig. 8c). Moving far from the fusion line decreased the effect of heat and grain size in the HAZ resulting in microstructure with fully finer acicular ferrite (see Fig. 8d).

A question might be raised on the different levels of pearlite in Figs. 7 and 8, as seen between the upstream pipe, downstream pipes and the elbow. This is explained here. The pearlite fraction in the microstructure of hypoeutectoid steels, similar to those of the pipes and elbow of this work, is directly related to the carbon content of the steel. Given that the carbon level in downstream pipe, elbow and upstream pipe are 0.104, 0.210, and 0.140%, respectively. Thus, the elbow which has the highest carbon level would have more pearlite than the pipes. The authors did not do any phase analysis, since it seemed quite far from the objectives of the work.

All the microstructures of the weld-metal cap, the root and the HAZ of the downstream pipe are normal and are expected with welding low carbon low alloy steels such as API X52. Thus, failure due to defective microstructure should be excluded from the root cause for the failure, but still the root profile as mentioned previously might have promoted such failure.

**Figure 9:**
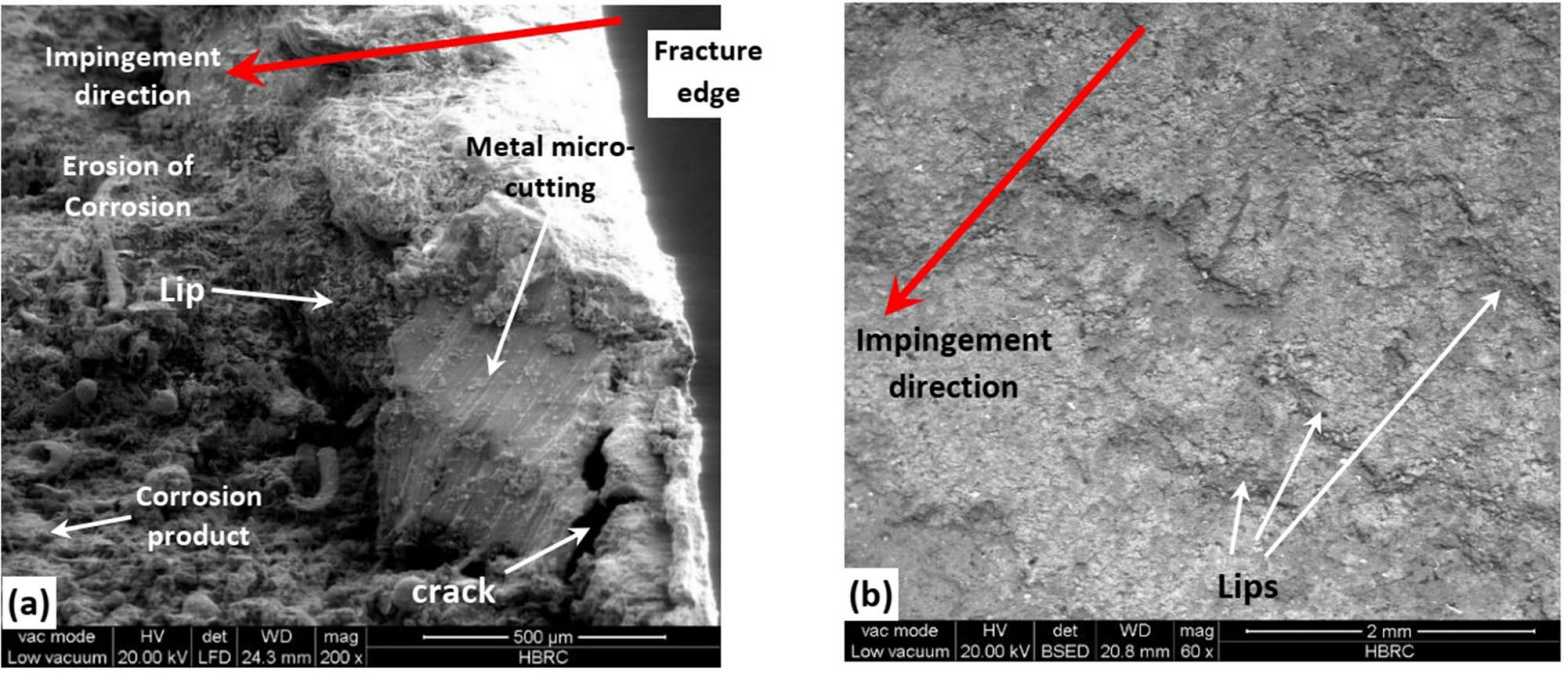
SEM micrographs for the fractured surface of the downstream pipe that show the effect of sand impingement (**a**) at, and (**b**) away from the fractured edge.

The fractography of the failed downstream pipe was further investigated using the scanning electron microscope (SEM) and energy dispersive X-ray spectroscopy (EDS). Figure 9 illustrates SEM investigation for the fractured surface of the downstream pipe at and away from the fractured edge. The SEM images were the best that can be taken from the failed samples. The failed sample was delivered for investigation after few months from removing from the flow line. This is very common for industrial failures. During this period, post failure corrosion occurred on the sample surfaces. The authors did their best to remove the corrosion products and succeeded partially in this. This is why the SEM images still show some corrosion products. Aiming at removal of all corrosion products might lead to damage of critical locations of the samples.

Obviously, sand impingement on the pipe surface due to turbulent flow caused several erosive wear effects (see Fig. 9a). It caused the formation of lips in the direction of impingement at and away from the fractured edge (see Fig. 9a and b). Pasha et al.^56^ reported that lips formation is usually due to tangential stress which leads to plastic deformation in the form of lips. Erosion and partially or even completely removal of the corrosion product or film was also demonstrated at some regions on the surface in the direction of sand impingement as shown in Fig. 9a. This indicates that the sand particles impacted the surface with different impact angles and velocities causing a variation in the erosion-corrosion rate. Furthermore, micro-cutting in the metal surface after removal of the corrosion film was observed near the fractured edge, which formed an adjacent crack in the direction of sand impingement (see Fig. 9a). This can be related to the severe erosion in such areas that removed both the corrosion film and layers from the metal surface and finally cracked the surface. Hence, sand impingement caused plastic deformation in the form of lips formation, metal micro-cutting, and cracks that implies cutting wear mechanism of failure.

**Figure 10:**
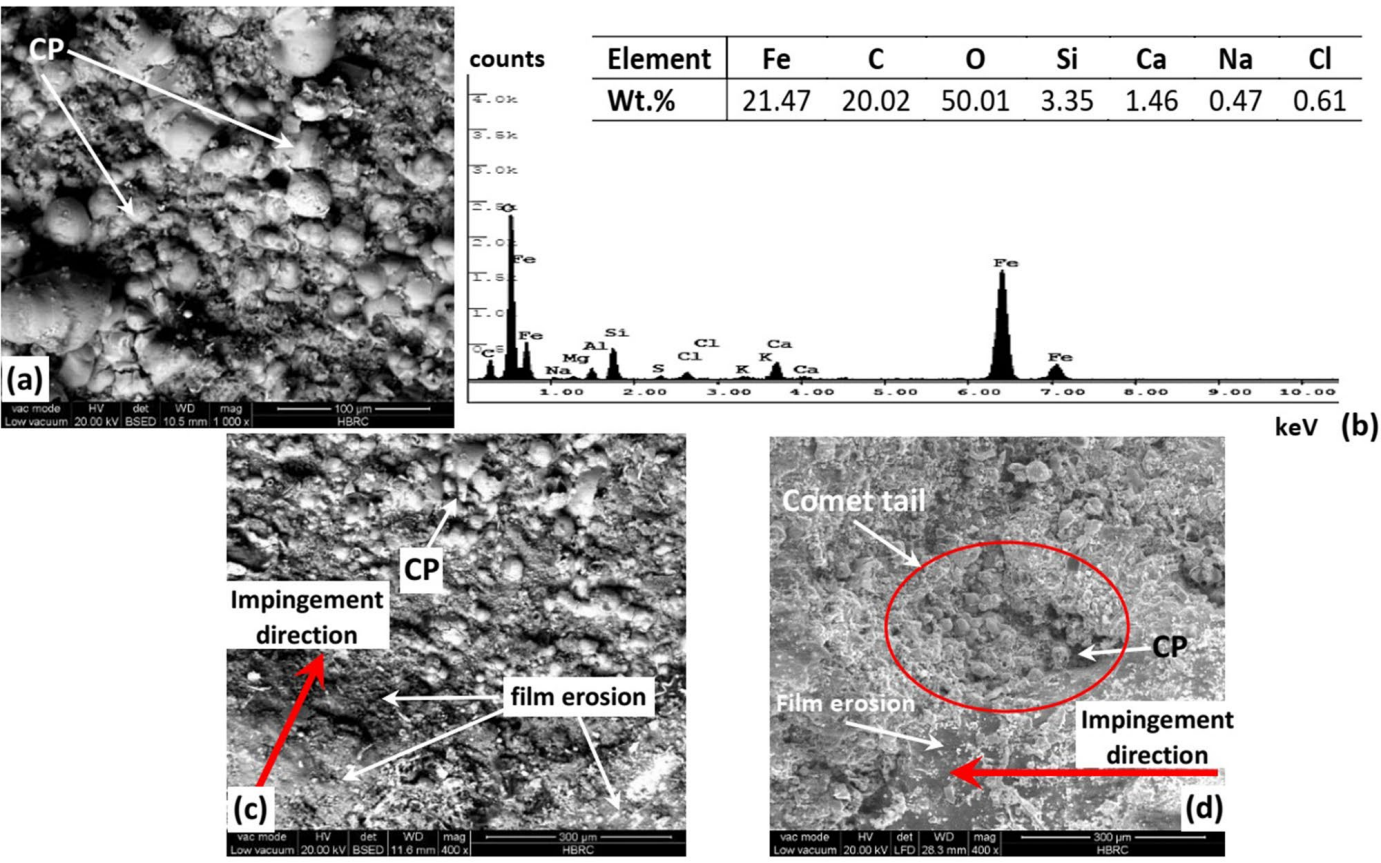
SEM micrographs for (**a**) the corrosion product (CP), (**b**) EDS analysis for the corrosion product, (**c**) erosion of the corrosion film, and (**d**) comet tail groove with corrosion product inside.

The corrosion product or film was further investigated using SEM and EDS analyses. Figure 10 demonstrates the SEM micrographs of the corrosion product (CP) at different locations and its EDS analysis. Figure 10a and b reveals the corrosion product morphology and its EDS analysis. The EDS analysis showed high levels of C, O and Fe, but lower levels of Ca, Na and Cl. Basically, rust (iron oxide and hydroxides), corrosion films (FeCO_3_, CaCO_3_), and lower levels of salt (NaCl) are expected. As mentioned earlier, FeCO_3_ film preferentially forms on the metal surface in the CO_2_ environment under the conditions of pH > 5 and temperature of 60 °C^22^, which are same as the present case. Also, the gas typically contains ~ 2 wt% CO_2_ and moisture of 75–160 PPMV that is essential for the formation of the bicarbonate ions (HCO_3_^-^) required for the formation of FeCO_3_ film. Thus, both the operating conditions and gas compositions promote the formation of the FeCO_3_ film.

However, the role of FeCO_3_ as protective or non-protective film depends on the degree of coverage or homogeneity on the surface^57^. Partial or homogenous coverage of the entire surface reduces the corrosion rate, but the roughness of the film can be preferential sites for stress concentrations and promotes localized corrosion^56,57^. Though, the formation of FeCO_3_ film on mild steel surface was reported to significantly reduce the corrosion rate^57^. Hence, FeCO_3_ film will be considered as protective film for the API 5L pipeline surface.

Figure 10c and d shows the erosion and removal of the FeCO_3_ film at different locations. Consequently, more active surface is exposed to be corroded and eroded again leading to higher material loss and more thinning in the pipeline. In order to explain the reason for such an event, several points should be taken into consideration; the sand impact angle and degree of film adhesion to the surface. Pasha et al.^56^ reported that the plastic deformation caused by sand impact at angle of 25° induced cutting wear mechanism of failure, while impact at 90° shows no evidence of erosion. Further, the adhesion of FeCO_3_ film to the surface depended on the steel microstructure^58^. The structure with ferrite embedded within pearlitic matrix shows better adhesion of the FeCO_3_ film to metal surface. When the ferrite within the pearlite matrix is dissolved by the medium, it allows the FeCO_3_ to deposit between the cementite plates and increases the adhesion with the metal surface. Thus, the microstructure of API 5L steel which demonstrated less content of pearlite embedded in the ferrite matrix will provide lower adhesion of the protective FeCO_3_ film to the surface. Concludingly, the erosion and removal of the protective FeCO_3_ film can be related to both the lower adhesion of the film to the surface and impact angle and velocity of sand induced by the turbulent flow.

Figure 10c illustrates formation of comet tails in the direction of sand impingement with corrosion product inside it. Clearly, the sand impingement removes the protective FeCO_3_ film and uncovers more active metal forming pit. Consequently, the electrolyte transfers inside the pit providing a stable condition for the initiation and growth of the pit and forms more corrosion product which is eroded again. The repetition of such process leads to higher material loss, more thinning of the pipeline and finally failure.

**Figure 11:**
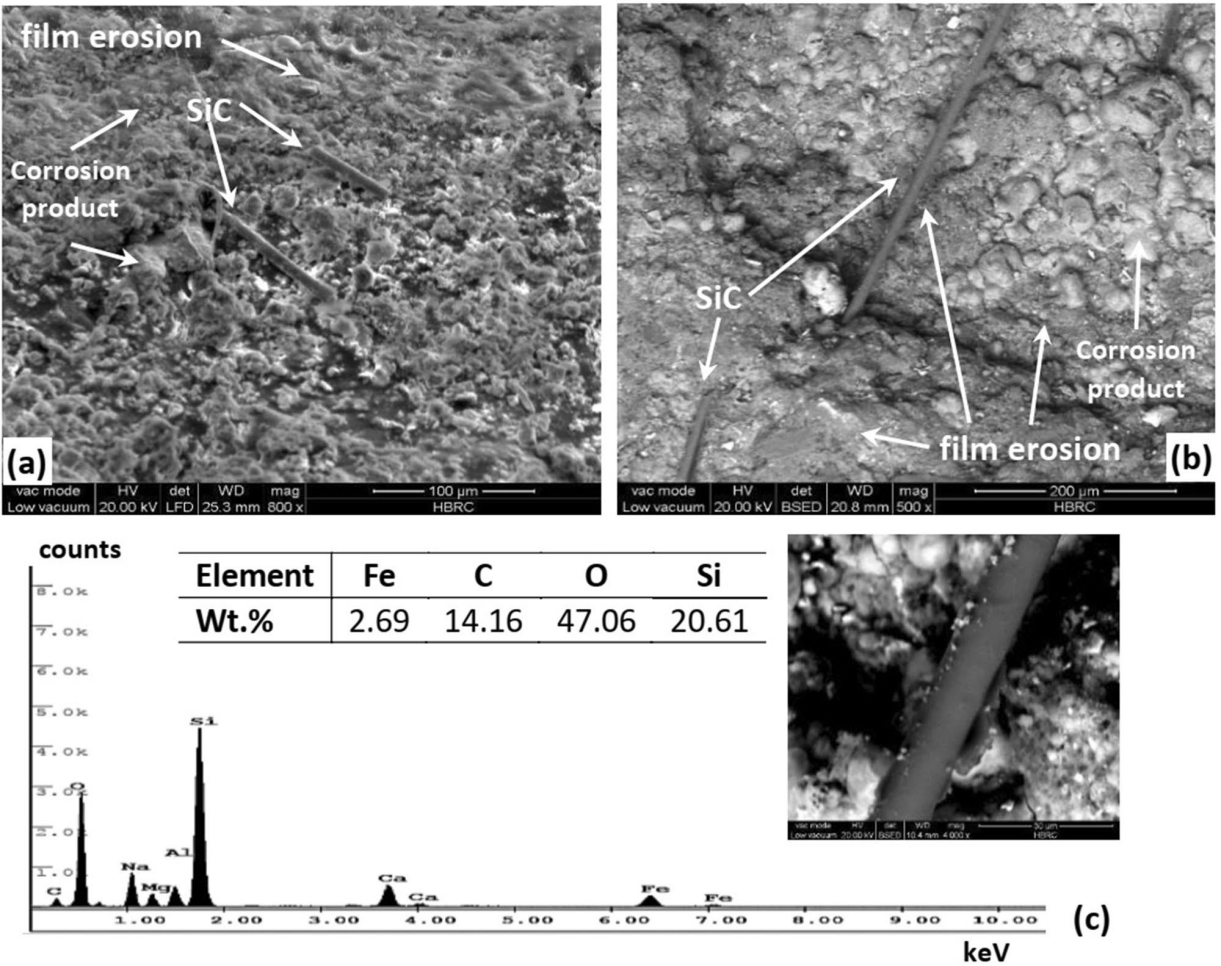
SEM micrographs for SiC fibers (**a**) over the corrosion film, (**b**) within the corrosion film, and (**c**) EDS analysis for the SiC fiber.

Moreover, the SEM investigation leads to the discovering of second phase particles embedded within the protective FeCO_3_ film. Figure 11 demonstrates SEM micrographs of the second phase particles and their EDS analysis. Figure 11a and b reveals that the second particles have morphology of fiber-like and located over and within the protective FeCO_3_ film. The EDS analysis of those second particles shows higher levels of Si, C, and O but lower level of Fe (see Fig. 11c). This can be related to the carbothermal reduction of silica and the formation of silicon carbides (SiC) along with carbon monoxide (CO). Lee et al.^59^ reported that silica can be reduced by carbon in gas form in the presence of iron as catalyst. They proposed the formation of SiC from silica through the following reactions:

1 $${\text{C }} + {\text{ CO}}_{{2}} \to {\text{2CO}}$$

2 $${\text{SiO}}_{{2}} + {\text{ CO}} \to {\text{SiO }} + {\text{ CO}}_{{2}}$$

3 $${\text{SiO }} + {\text{ 2C}} \to {\text{SiC }} + {\text{ CO}}$$

This explains the higher levels of Si, C and O which are related to the formation of both SiC and CO.

Also, they reported that the reduction takes place during the carbothermal reduction of iron oxide. Thus, the formation of SiC is highly promoted during the formation of the protective FeCO_3_ film. Hence, the reason for the presence of SiC embedded in the protective FeCO_3_ film is now verified. Furthermore, the removal of the protective FeCO_3_ film via erosion led to completely or partially uncovering the SiC as shown in Fig. 11a and b. However, the formation of SiC within the protective film might retain the protective FeCO_3_ film through reinforcement, but unfortunately the content of SiC was not enough to do so. Thus, the protective FeCO_3_ film was eroded and removed by the sand impingement.

The quantitative investigation of erosion and the kinetics of corrosion are quite possible in the planned corrosion studies. However, in the current investigation, where only the final state of the damage can be assessed and investigated, it is almost impossible to clearly identify the operating mechanisms of erosion and the kinetics of corrosion. These can change during the process of damage and several mechanisms can operate at certain stages of the damage. These cannot be estimated from the failed specimen. Any effort in this direction would be, at best, a speculation of the operating mechanisms and kinetics of damage. For this reason, the authors did not include any speculation of the erosion mechanisms and corrosion kinetics.

### Mechanical properties

The mechanical properties of the downstream pipe, elbow and upstream pipe were investigated to ensure that their materials had satisfied the properties. Both tensile test and Vickers hardness test were conducted on the material of the downstream pipe, elbow and upstream pipe and the measurements are demonstrated in Table 3. The obtained tensile properties are compared to the standard values for grade X52 according to API 5L standard^27^. The tensile properties of the downstream pipe, elbow and upstream pipe satisfy the requirements for grade API 5L X52. As for the hardness test, it is not required for the acceptance of the pipe material according to API 5L standard. Though, the hardness measurements agree with other researchers for grade X52 carbon steel^60^.

**Table 3:** The mechanical properties of the downstream pipe, elbow, and upstream pipe along with that of acceptable properties for grade X52 according to API 5L standard.

Property	0.2% offset yield strength, MPa	Tensile strength, MPa	Elongation, %	Required elongation, %	Hardness, HV
Downstream pipe	466 ± 10	554 ± 10	20 ± 5	17.1 (min)	201 ± 2
Upstream pipe	441 ± 10	581 ± 10	17 ± 5	15.9 (min)	211 ± 2
Elbow	385 ± 10	535 ± 10	28 ± 5	18.9 (min)	179 ± 3
API 5L-X52^27^	360 (min)	460 (min)	Calculated based on tensile test sample cross sectional area and UTS		–

The higher tensile strength of the upstream pipe compared with the downstream pipe is explained by the higher levels of C and Mn in the upstream pipe. These elements have direct effect on the tensile strength of carbon steels. It is worth mentioning here that the mechanical properties of the welded area were not investigated in this work since the failure occurred in the pipe base metal and not in the weld zone.

As mentioned earlier, the banded structure as that of the elbow microstructure causes anisotropy in some of the mechanical properties^51^. It has been reported that the banded structure of hot rolled steel has no significant effect on yield strength, tensile strength, and hardness but more significant effect on the impact toughness^60^. Banded structure in certain circumstances might not be the preferred path for crack propagation in mechanical failures. Hence, there is no need to encounter the anisotropy effect on the mechanical properties with our measurements^61^. Concludingly, the mechanical properties of the downstream pipe, elbow and upstream pipe are acceptable and are not considered as a root cause for failure.

## Conclusions and preventive actions

A detailed failure analysis of API X52 high strength carbon alloy steel pipeline was conducted. The pipeline includes the upstream pipe, elbow, and the downstream pipe. The investigation found that the main failure occurred in the downstream pipe located near the welded joint at the elbow outlet instead of elbow, which was interesting. The main mechanism of failure was found to be erosion-corrosion mechanism that caused breakdown of the protective FeCO_3_ film, thinning of the downstream pipe, and finally failure. It is believed that erosion-corrosion is induced by sand impingement due to turbulent flow that was promoted by sudden change in the flow cross section between the elbow inlet and upstream pipe as a result of the poor welding quality of joint at the elbow outlet.

Consequently, it is recommended to impose more control to sand screen to reduce the probability for the presence of sand particles in the flow to the minimum. Selection of proper materials that are more resistant to erosion-corrosion and offer strong adhesive protective film and fast recovery of the film if worn off can also be considered. Serious control during the welding procedure to keep the root penetration at minimum level (1.6 mm) and its sound profile can contribute to minimize the failure risk. Finally, using pipes and elbows with the same wall thickness will prevent the sudden change in the flow cross section and in turn reduces the risk of turbulent flow.

## Data Availability

All data generated or analyzed during this study are included in this published article.
